# Notes and News

**Published:** 1905-06-03

**Authors:** 


### Notes and News.

The Prince and Princess of Wales, Grand President and Lady Grand President of the League of Mercy, will receive the Presidents, Lady Presidents, and certain others connected with the League cn the afternoon of Saturday, July 1st, at Marlborough House.

The Copenhagen Port authorities have withdrawn the order declaring Leith to be plague-infected.

Electric lighting and an electric lift have been added to the Metropolitan Hospital. The cost has been defrayed through a bequest from the late A. Ocran Crooke.

An inquiry has been made as to the fitness of calves to be used for food after vaccination at the Local Government Board stations. The reply is that the calves are not rendered unfit for consumption, and it is not therefore proposed to destroy them.

Before Mrs. Choate, the wife of the retiring American Ambassador, sailed for the United States on Tuesday, she sent a donation of £20 as a parting gift to the Hospital for Sick Children, Great Ormond Street, in which institution she was much interested.

A new vegetable has just been introduced to us from Japan. This is the bracken of our woods and moorlands, which, we are told, when properly prepared, is very like asparagus. If found suitable to English digestions it will be an inexpensive addition to the table.

According to the report of the Medical Officer of Health for the City of London, it appears that in the matter of milk adulteration foreigners are the worst offenders. One in nine samples sold by foreigners was adulterated, against one in 16 sold by British shopkeepers.

The Conway and Penmaenmawr rural authorities are about to build a joint isolation hospital.

The Ebbw Vale Hospital, Monmouthshire, is to be extended. It was opened four years ago.

It is proposed to erect a new hospital for Hounslow. A site on the Staines Road has been selected.

Inquests have been held over two children who have died at Stoke Newington. In both cases the children had been given a proprietary preparation known as "Liquozone."

It is intended to establish a hospital between Kingussie and Kincie to provide for the district between Lochaber March to the river Findhorn at Tomartin. The cost is estimated at £3,472.

A special performance of "Lady Windermere's Fan" was announced for June 1st at the Borough Theatre, Stratford, in aid of the West Ham and East London Hospital.

A large meeting to further the provision of a sanatorium for consumptives for Dumfriesshire was held last week. It was announced that nearly £7,000 had been promised.

Sir Horace Brooks Marshall, J.P., will preside at the Anniversary Festival of the Newsvendors' Benevolent and Provident Institution, at De Keyser's Royal Hotel, on October 24.

The Metropolitan Asylums Board are forwarding for the approval of the Local Government Board a scheme to provide suitable accommodation for tuberculous children at Rustington, near Littlehampton.

The foundation-stone has been laid of a new Convalescent Home for the Durham County Hospital. The cost of building and site is estimated at £7,000, of which £5,000 has already been subscribed.

The Lord Mayor has kindly consented to preside at a meeting at the Mansion House, on July 13th, to commemorate the 21st anniversary of the opening of the Home by the present King and Queen.

The plans of the New King's College Hospital are on view at Carpenter's Hall, London Wall. The designs have been carried out under the instructions of the hospital authorities by Mr. W. Pite, F.R.I.B.A. They are the result of a most careful study of the essential features to secure economy and efficiency of administration.

The Steyning guardians are about to erect new workhouse infirmary buildings. No fewer than 23 contractors submitted offers. The lowest estimate came out at a cost of about £150 per bed, but a guardian considered the matter should be referred back for a reduction of the proposed expenditure. Another guardian pointed out that £150 per bed was a very reasonable cost in the present day.

